# The PyPIF5-*PymiR156a*-PySPL9-PyMYB114/MYB10 module regulates light-induced anthocyanin biosynthesis in red pear

**DOI:** 10.1186/s43897-021-00018-5

**Published:** 2021-11-03

**Authors:** Hainan Liu, Qun Shu, Kui Lin-Wang, Andrew C. Allan, Richard V. Espley, Jun Su, Maosong Pei, Jun Wu

**Affiliations:** 1 College of Horticulture, State Key Laboratory of Crop Genetics and Germplasm Enhancement, Nanjing Agricultural University, Nanjing, 210095 China; 2 College of Horticulture and Plant Conservation, Henan University of Science and Technology, Luoyang, 471023 China; 3 Institute of Horticulture, Yunnan Academy of Agricultural Sciences, Kunming, 650205 China; 4 The New Zealand Institute for Plant & Food Research Limited, Auckland, New Zealand; 5 School of Biological Sciences, University of Auckland, Auckland, New Zealand

**Keywords:** Red-skinned pear, Anthocyanin, PyPIF5, *PymiR156a*, PySPL9, PyMYB114/MYB10, PyMYB10 (Pbr016663.1); PyMYB114 (Pbr042924.1); PyPIF5 (Pbr029607.1); PySPL9 (Pbr019232.1); PybHLH3 (Pbr017379.1); PyERF3 (Pbr030451.1).

## Abstract

Some cultivars of pear (*Pyrus* L.) show attractive red fruit skin due to anthocyanin accumulation. This pigmentation can be affected by environmental conditions, especially light. To explore the light-induced regulation network for anthocyanin biosynthesis and fruit coloration in pear, small RNA libraries and mRNA libraries from fruit skins of ‘Yunhongyihao’ pear were constructed to compare the difference between bagging and debagging treatments. Analysis of RNA-seq of fruit skins with limited light (bagged) and exposed to light (debagged), showed that *PyPIF5* was down-regulated after bag removal. *PymiR156a* was also differentially expressed between bagged and debagged fruit skins. We found that PyPIF5 negatively regulated *PymiR156a* expression in bagged fruits by directly binding to the G-box motif in its promoter. In addition, *PymiR156a* overexpression promoted anthocyanin accumulation in both pear skin and apple calli. We confirmed that *PymiR156a* mediated the cleavage of *PySPL9*, and that the target PySPL9 protein could form heterodimers with two key anthocyanin regulators (PyMYB114/PyMYB10). We proposed a new module of PyPIF5-*PymiR156a*-PySPL9-PyMYB114/MYB10. When the bagged fruits were re-exposed to light, PyPIF5 was down-regulated and its inhibitory effect on *PymiR156a* was weakened, which leads to degradation of the target *PySPL*, thus eliminating the blocking effect of PySPL on the formation of the regulatory MYB complexes. Ultimately, this promotes anthocyanin biosynthesis in pear skin.

**Supplementary Information:**

The online version contains supplementary material available at 10.1186/s43897-021-00018-5.

## Core

A novel regulatory module PyPIF5-*PymiR156a*-PySPL9-PyMYB114/MYB10 underlying the light-induced anthocyanin biosynthesis in red pear is unraveled. Light down-regulates *PyPIF5*, which releases *PymiR156a* from PyPIF5’s inhibition and allows *PymiR156a* to degrade its target *PySPL* transcripts, promoting the formation of the PyMYB114/MYB10 complexes that increase the anthocyanin biosynthesis.

## Introduction

Pear (*Pyrus* L.) is an important temperate deciduous fruit crop. Compared to pears with green or brown (russetted) skins, red skinned varieties are popular but relatively scarce. Pigmentation of most fruits begins at the middle or late stages of fruit development, with coloration partly determined by environmental influences (Yang et al. 2013), especially light. Light is generally necessary for anthocyanin biosynthesis and organ coloration in various plants (Azuma et al. 2012; Feng et al. 2013; Jiang et al. 2017). In previous studies, MYB (v-myb myeloblastosis viral oncogene homolog), bHLH (Basic Helix-Loop-Helix) and WDR (WD40-repeat) were found to be the key transcription factors (TFs) that regulate anthocyanin biosynthesis, binding to the promoters of genes that encode enzymes of the anthocyanin biosynthetic pathway Espley et al. 2007, Toledo-Ortiz et al. 2003 (Xu et al. 2015). These TFs form the MBW transcription complex, such as found in *Arabidopsis* (Xu et al. 2013), Petunia (Quattrocchio et al. 1993), and apple (An et al. 2012; Liu et al. 2017). Besides the MBW complex, a new PybHLH3-PyMYB114-PyERF3 transcription complex was found in pear (Yao et al. 2017). Other regulatory factors have also been reported, such as WRKY (An et al. 2019; Li et al. 2020), NAC (Zhou et al. 2015) and HB (HD-Zip) (Jiang et al. 2017). In addition, some light-responsive TFs have been found to affect anthocyanin biosynthesis by regulating genes that encode enzymes of the pathway, such as HY5 (An et al. 2017), BBX (Bai et al. 2019; Xu et al. 2016) and bZIPa (Liu et al. 2019).

While anthocyanin biosynthesis is regulated at the transcriptional level, the expressed genes are also affected by RNA processing (post-transcriptional regulation) (Cui et al. 2014; Gou et al. 2011; Yang et al. 2019). MicroRNA (miRNA) plays an important role in post-transcriptional gene regulation. Multiple light response elements were found in miRNAs (Gou et al. 2011) and increasing studies have shown that miRNAs, along with their downstream targets, play important roles in development, growth and other important life processes in plants (Cui et al. 2020; Guo et al. 2019; Miao et al. 2019; Yu et al. 2019). Some of these miRNA-mediated processes overlap with anthocyanin biosynthesis, such as *miR156* targeted *AtSPL9* which acts as a negative regulator of anthocyanin biosynthesis in *Arabidopsis* (Gou et al. 2011). Stress-induced anthocyanin accumulation in *Arabidopsis* is regulated by the *miR156-*SPLs-DFR pathway (Cui et al. 2014). *MiR156a* also plays an important role during light-induced anthocyanin biosynthesis in apple skin, and was shown by using lncRNA MLNC3.2 and MLNC4.6 as endogenous target mimics, to prevent cleavage effects of *miR156a* on *SPL2*-like and *SPL33* mRNA (Yang et al. 2019). These results suggested that the regulatory pathway of miR156 was different across diverse species. To date, the miR156 regulatory pathway in red pear is unknown. Furthermore, other miRNA family members and their corresponding targets are involved in the regulation of anthocyanin biosynthesis, such as miR399d/PHO2 (Peng et al. 2020), miR858/MYB (Jia et al. 2015) and miR828/MYB (Yang et al. 2013). Research tends to focus on the relationship of miRNA-target genes and how miRNAs exert function through these genes. There are fewer studies on miRNA regulatory factors. In regard to the process of light-induced anthocyanin biosynthesis, the upstream regulatory mechanism of anthocyanin associated-miRNAs has not yet been reported.

In this study, we compared the transcriptome and small RNA data of bagged and debagged pear fruit skins, providing new data for the exploration of the miRNA regulated light-induced anthocyanin biosynthesis pathway and the discovery of the upstream regulator of miRNAs. Our study attempts to establish a regulation model of upstream regulator-miRNA-target-transcription factor of structural genes under light-induced condition.

## Results

### Identification of DE-miRNAs and DEGs

**Fig. 1:**
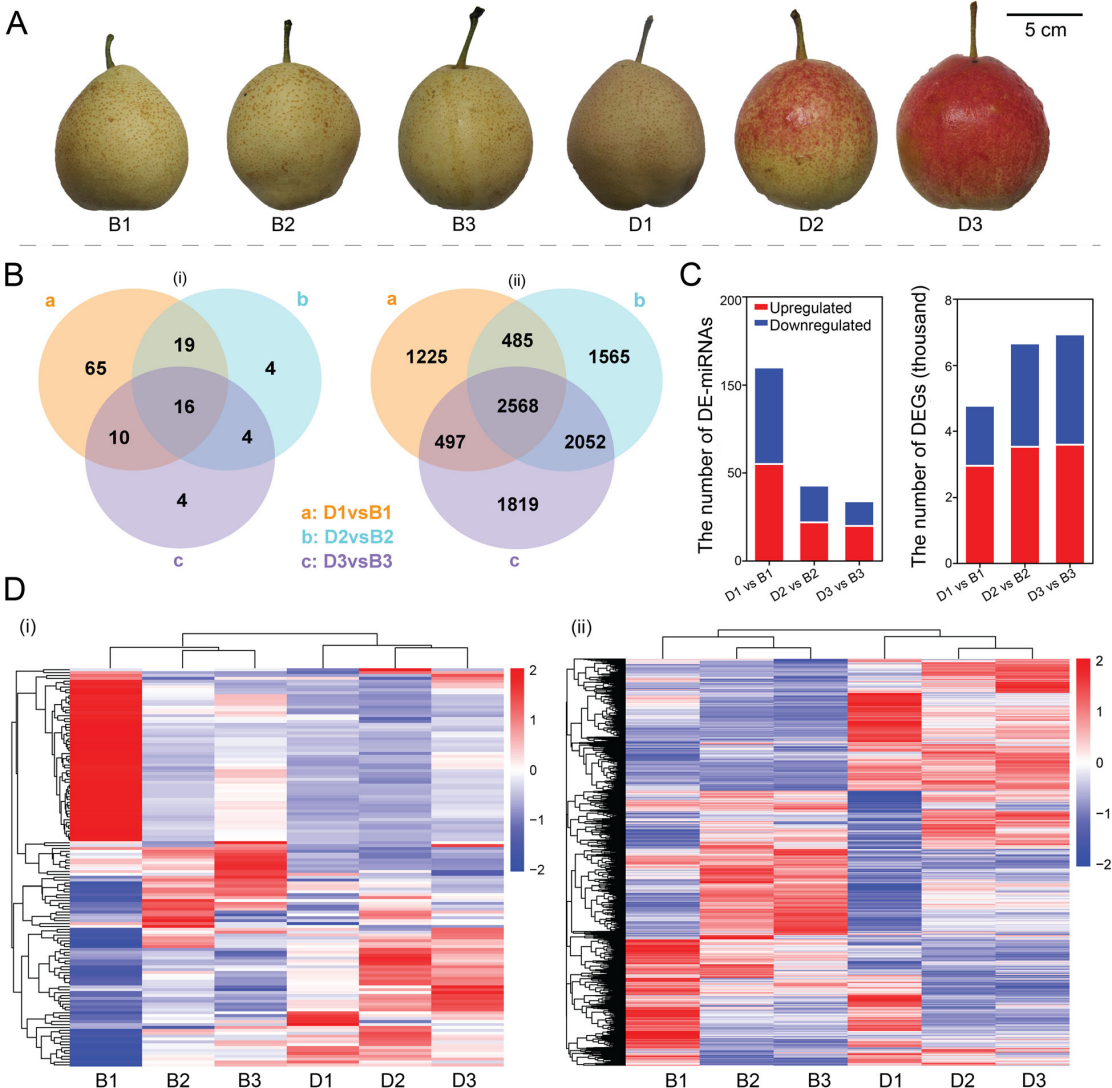
Phenotype of “Yunhongyihao” and sequencing of sRNAs and RNA in bagged and debagged fruits. **A** Phenotype of “Yunhongyihao” at 4, 8, and 10 days after bag removal for bagged (B1, B2, B3) and debagged (D1, D2, D3) fruits. **B** Venn diagram showing the number of DE-miRNAs (i) and DEGs (ii). **C** The number of upregulated and downregulated DE-miRNAs and DEGs. **D** Cluster analysis of the expression levels of DE-miRNAs (i) and DEGs (ii)

Anthocyanin accumulated rapidly in the pear fruit skin at 4 (D1), 8 (D2), and 10 (D3) days after bag removal, but no anthocyanin accumulation was observed in the corresponding days with bagged fruits (B1, B2, B3) (Fig. 1A). To examine miRNAs levels, we performed small RNAs (sRNAs) sequencing from pear skins of bagged (B1, B2, B3) and debagged (D1, D2, D3) fruits. After removing low-quality reads and adapter sequences, clean reads were used for identification of miRNAs. A total of 34 conserved miRNA families were identified. In addition, there were 110, 43 and 34 differentially expressed (DE)-miRNAs in bagged (B1, B2, B3) and debagged fruit skins (D1, D2, D3) (Fig. 1B). A total of 55, 22 and 20 DE-miRNAs were upregulated and 55, 21 and 14 were downregulated in comparisons of D1/B1, D2/B2 and D3/B3 (Fig. 1C).

To investigate the expression profile of miRNAs-targeted genes, RNA sequence analysis was performed using corresponding samples. An average of 9 billion clean bases was generated for each sample. There were 4775, 6670, 6936 differentially expressed genes (DEGs) identified in bagged and debagged fruit skins (Fig. 1B) at the three time points, with 2957, 3529 and 3596 upregulated and 1818, 3141 and 3340 downregulated in D1/B1, D2/B2 and D3/B3 (Fig. 1C). Furthermore, a cluster analysis of DE-miRNAs and DEGs showed that bagging and debagging treatments caused dramatic change in expression levels of miRNAs and mRNAs, and bagged and debagged fruits can be well divided according to their expression profiles (Fig. 1D).

Based on the functional enrichment analysis for predicted targets and DEGs (Fig. S1A-D), 10 conserved DE-miRNAs from 6 miRNA families (including miR858, miR408, miR399, miR164, miR172 and miR156) and 628 DEGs from 23 family (such as *COP*, *PIF*, *bZIP*, *MYB*, *SPL*, *NAC*, etc.) were selected as candidate regulators involved in light-induced anthocyanin regulation. In addition, 329 predicted target genes of candidate DE-miRNAs were overlapped with genes differentially expressed in at least one comparison (Fig. S1E). The predicted targets of *PymiR156a* had the highest overlap ratio with DEGs, which indicated that *PymiR156a* had significant regulation on target genes, and may play an important regulatory role during light-induced anthocyanin biosynthesis.

Histochemical analysis of β-glucuronidase (GUS) expression driven by *PymiR156a* promoter indicated that the *PymiR156a* promoter had the ability of initiating gene expression of *GUS* reporter gene (Fig. S2). Furthermore, we analyzed promoter region of pre-miR156a (the genomic region ~ 3000-bp upstream of *PymiR156a* precursor) using the PlantCARE online software (http://bioinformatics.psb.ugent.be/webtools/plantcare/html/). There were 17 light-responsive elements in the pre-miR156a promoter, including one AE-box, three Box 4, five G-boxes, two GATA-motifs, two GT1-motifs, one I-box, one LAMP-element, one sp1 element, and one TCCC-motif (Supplementary Table S1). Among these elements, multiple typical PIF-binding sites (G-box motif) were found in the *PymiR156a* promoter. Interestingly, we found a phytochrome-interacting transcription factor, *PyPIF5*, was down-regulated by bag removal, and the expression of *PyPIF5* and *PymiR156a* showed a negative correlation, suggesting that there may be a regulatory relationship between them.

### PyPIF5 suppresses *PymiR156a* expression by binding to a G-box in its promoter

**Fig. 2:**
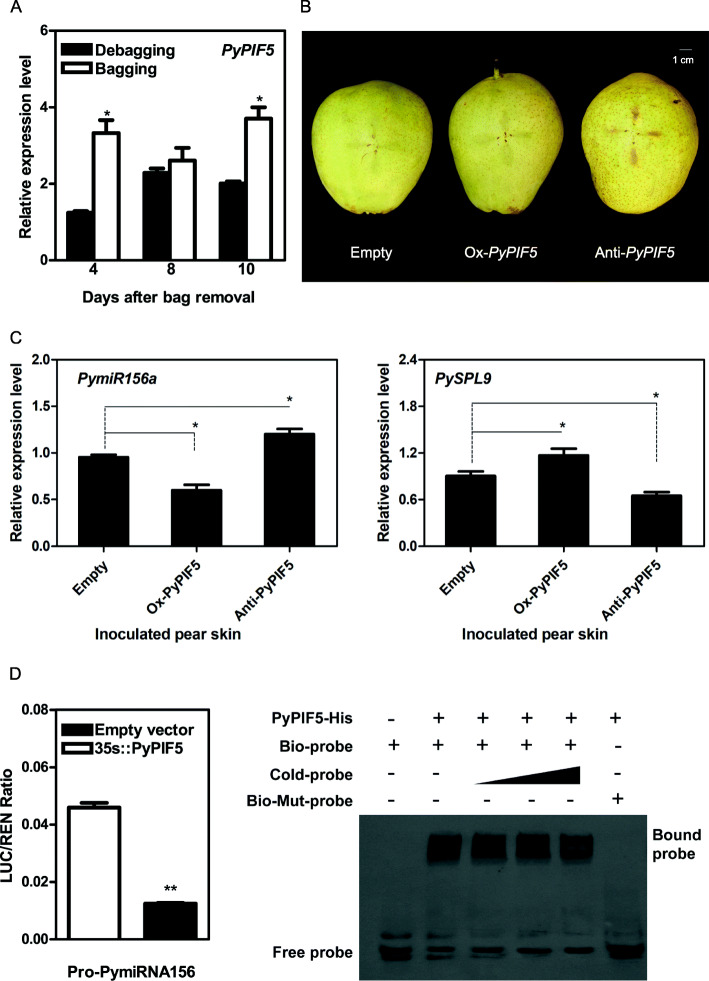
PyPIF5 protein suppresses *PymiR156a* expression by binding G-box in promoter. **A** The expression profile of *PyPIF5*. **B** Overexpression and suppression of *PyPIF5*. **C** Expression pattern analysis *PymiR156a* and *PySPL9* in inoculated pear. **D** Dual-luciferase assay for the transcriptional activation of PyPIF5. **E** Electrophoretic mobility shift assay for examining the DNA-binding property of PyPIF5. As the progressively increase of Cold probe concentration, the accumulation of Free probe increases. The addition of mutant probes further confirmed that PyPIF5 can specifically bind to the G-box sequence within *PymiR156a* promoter

PIFs belong to the basic helix-loop-helix (bHLH) family with a typical highly conserved APB domain [active phytochrome B (phyB) binding] (Fig. S3). We found *PyPIF5* was down-regulated in debagged fruits (Fig. 2A). Furthermore, multiple PIF-binding sites were found in the *PymiR156a* promoter (Supplementary Table S1), which suggested that the down-regulated *PyPIF5* may be involved in expression suppression of *PymiR156a*. Therefore, we focus on the regulatory relationship of PyPIF5 to *PymiR156a*. For its functional verification, we suppressed and overexpressed *PyPIF5* in “Zaosu” pear skins by using agrobacterium-mediated transient expression method, and observed that the suppression of *PyPIF5* could produce the red patches on pear skins, while no visible red patches in overexpression treatment or empty vector control (Fig. 2B). The qRT-PCR analysis of the infiltrated pear skin patches showed upregulation of *PymiR156a* in *PyPIF5* overexpression patches and downregulation of *PymiR156a* in *PyPIF5* suppression patches, and the expression pattern of *PySPL9* was negatively correlated with that of *PymiR156a* (Fig. 2C). These above results show that alteration of *PyPIF5* expression affected anthocyanin production with associated changes of *PymiR156a* and *PySPL9*. Dual-luciferase assays suggested that the co-expression of PyPIF5 strongly suppressed the *LUC* reporter gene activities when driven by *PymiR156a* promoter, indicating the inhibitory effect of PyPIF5 on *PymiR156a* promoter (Fig. 2D).

The regulatory relationship of PyPIF5 to *PymiR156a* was further tested by Electrophoretic mobility shift assay (EMSA). We expressed and purified recombinant PyPIF5-His fusion protein and prepared *PymiR156a* promoter probe containing G-box motif (and its corresponding mutated sequence) (Fig. S4), to test whether PyPIF5 protein could directly interact with the *PymiR156a* promoter in vitro*.* EMSA demonstrated that PyPIF5 bound to the oligonucleotide sequence of the G-box in *PymiR156a* promoter, and competitive reactions (with cold probe competitor and mutated probe competitor) showed that the binding reaction was specific. PyPIF5 directly bound to the biotin labeled probes. This binding activity was gradually reduced upon the adding of unlabeled cold probe. Furthermore, mutant versions of probes with mutations destroying the G-box motif showed no binding of PyPIF5. All together, these results demonstrated that PyPIF5 directly binds to the *PymiR156a* promoter, which is consistent with the observation that PyPIF5 regulates *PymiR156a* expression abundance (Fig. 2E). Thus, we concluded that PyPIF5 protein could specifically bind to G-box motif in the *PymiR156a* promoter and repress its expression.

### Target gene prediction and genome-wide analysis of miR156 targets

**Fig. 3:**
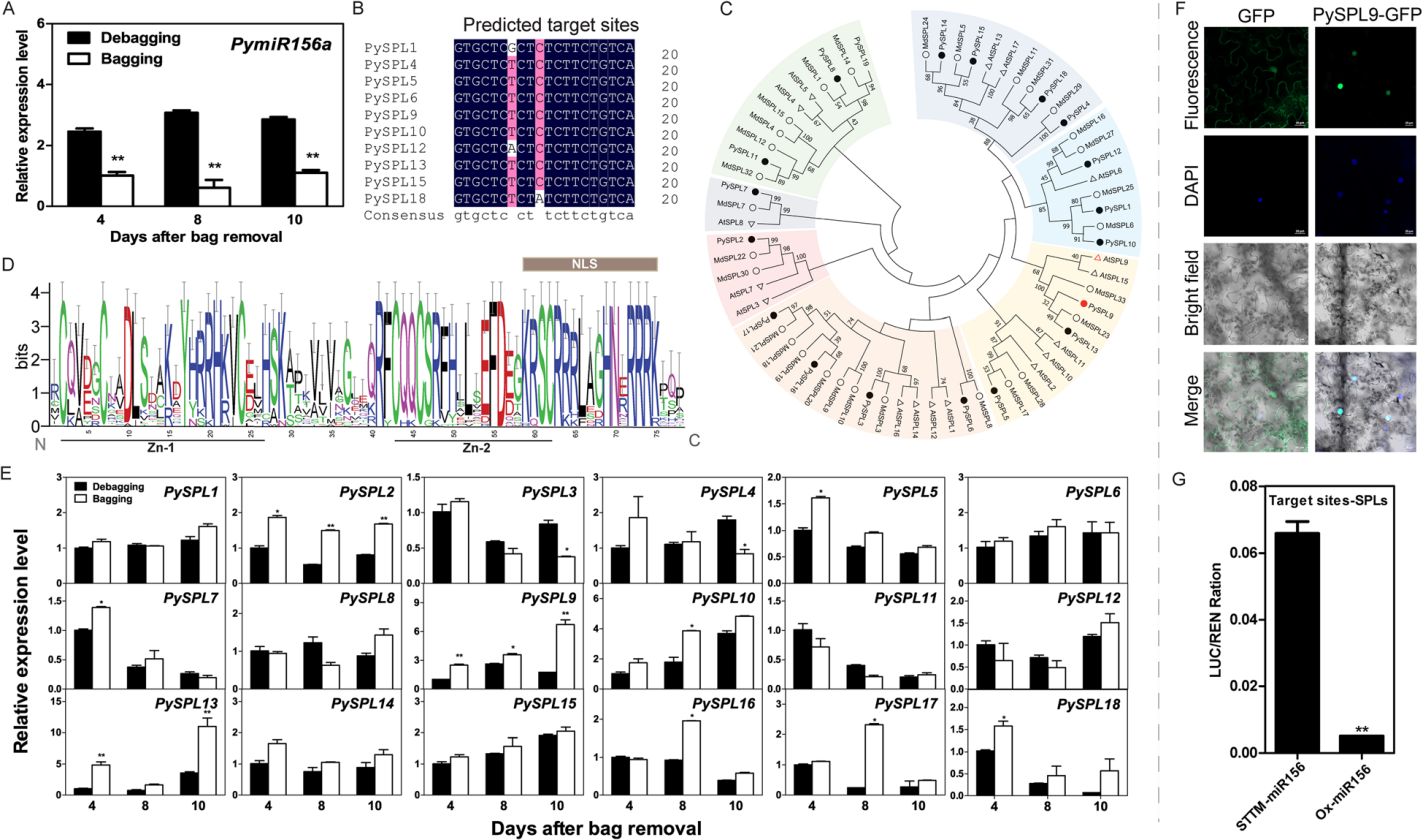
The expression profile of *PymiR156a* and its targets analysis. **A** The expression profile of *PymiR156a* in bagged and debagged fruits skins. **B** Predicted target sites on *PySPLs* of *PymiR156a*. **C** Phylogenetic analysis of SPLs from pear, apple and *Arabidopsis thaliana*. **D** Sequence logo of the SBP domain of PySPL proteins (by weblogo.berkeley.edu). The conserved zinc finger structures (Zn-1 and Zn-2) and the nuclear localization signal (NLS) are indicated. **E** Expression profile analysis of *PySPLs*. **F** Subcellular localization of PySPL9 protein. **G** Verification of cleavage events of *PymiR156a* on *PySPL*

Compared with bagged fruits, the expression of *PymiR156a* significantly increased in debagged fruits (Fig. 3A). Squamosa promoter binding protein-like (SPL) family members were predicted as *PymiR156a*-targets (Fig. 3B) by using the psRNATarget server (Dai and Zhao 2011). In total, 19 *PySPL* genes were identified in the pear genome. A phylogenetic tree was constructed using SPL amino acid sequences from pear, apple and *Arabidopsis* (by using neighbor-joining algorithm). These SPL proteins were clustered into seven groups, and PySPLs (marked with black solid dots) were distributed in seven cluster groups (Fig. 3C). All PySPL members have a conserved SBP domain, containing two zinc finger-like structures (Zn-1 and -2) and a highly conserved nuclear localization signal (NLS) (Fig. 3D).

In contrast to the *PymiR156a* expression pattern, we observed a significant decrease in the expression levels of *PySPL2*, *5*, *7*, *9*, *10*, *13*, *16*, *17* and *PySPL18* at least in one comparison (D1/B1, D2/B3, or D3/B3) (Fig. 3E). The predicted protein sequence of PySPL9 has high sequence similarity to *A. thaliana* AtSPL9 (shown in Fig. 3C), which was reported to act as a negative regulator of anthocyanin biosynthesis (Gou et al. 2011). To examine the subcellular localization of PySPL proteins, a PySPL9-GFP fusion construct was generated and transferred into tobacco by *Agrobacterium*-mediated transformation. Green fluorescence signals were observed specifically in the nucleus by the co-localization with DAPI (4′,6-Diamidino-2-Phenylindole) staining. The result confirms that PySPL9 protein is a nuclear protein (Fig. 3F), which is consistent with the identification of an NLS in the SBP domain.

Effector vectors were constructed to express the pre-miR156a (Ox-miR156a) and short tandem target mimic-miR156a (STTM-miR156a) structure, and a reporter vector containing *LUC* (firefly luciferase) gene fused to *PymiR156a* target site sequences (Target site-SPLs). After co-infiltration into *Nicotiana benthamiana* leaves, the target site-SPLs and STTM-miR156a effector vector showed a significantly higher LUC/REN ratio than that of Ox-miR156a (Fig. 3G). These results show that the predicted target sites in pear SPL mRNA can be identified and cleaved by *PymiR156a*.

### Overexpression of *PymiR156a* promotes anthocyanin accumulation

**Fig. 4:**
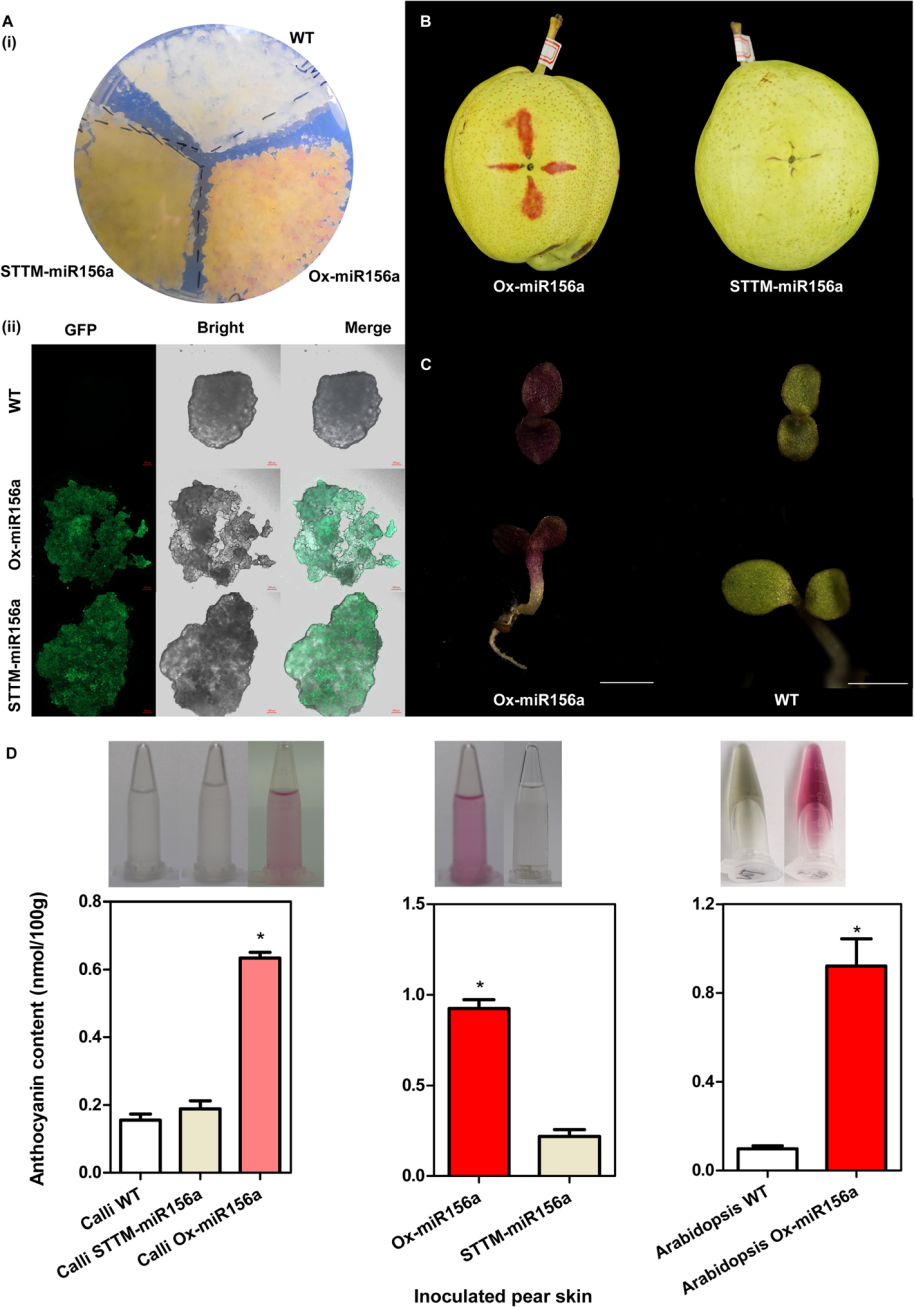
Overexpression and suppression of *PymiR156a.* **A** Overexpression and suppression of *PymiR156a* in apple calli (i), and Green fluorescence detection (ii). **B** Overexpression and suppression of *PymiR156a* in Cv. “Zaosu”. **C** Overexpression of *PymiR156a* in *Arabidopsis* seedling. **D** Anthocyanin content in apple calli, inoculated pear skin and *Arabidopsis* seedling (nmol/100 mg fresh weight)

To investigate the function of *PymiR156a*, we transformed overexpression (Ox-miR156a) and suppression (STTM-miR156a) constructs (also fused to Green fluorescent protein reporter, GFP) into apple calli through *Agrobacterium*-mediated genetic transformation according to An et al. (2017). The *PymiR156a* overexpression calli turned red, while WT control and *PymiR156a* suppression calli did not (Fig. 4A). GFP detection in apple calli showed that the GFP was expressed both in overexpression and suppression calli (Fig. 4A), which verifies successful transformation of the apple calli. Transient fruit transformation was also performed to the pear cultivar “Zaosu”, and the same phenotypes were also observed (Fig. 4B). We also observed anthocyanin accumulation in *PymiR156a* overexpression *Arabidopsis* seedlings, which promoted the cotyledon changed into red (Fig. 4C). Furthermore, the anthocyanin content determination of apple calli, inoculated pear skin and *Arabidopsis* seedling confirmed the accumulation of anthocyanin caused by *PymiR156a* (Fig. 4D) These results suggested that *PymiR156a* plays a positive role in anthocyanin biosynthesis.

To further illustrate the regulation between *PymiR156a* and *PySPL9*, co-expression *PymiR156a* and *PySPL9*, by mixing them using agrobacterium-mediated transformation in “Zaosu” pear were conducted. We observed that overexpression of *PymiR156a* alone promoted anthocyanin accumulation (Fig. 4), but not when co-expressed with *PymiR156a* and excessive *PySPL9* (Fig. S5), which demonstrated that the effect of *PymiR156a* overexpression on pigment accumulation was blocked by increasing the abundance of *PySPL9* in the fruit skin.

### Interaction between *PymiR156a*-targeted PySPL9 and PyMYB114/PyMYB10

**Fig. 5:**
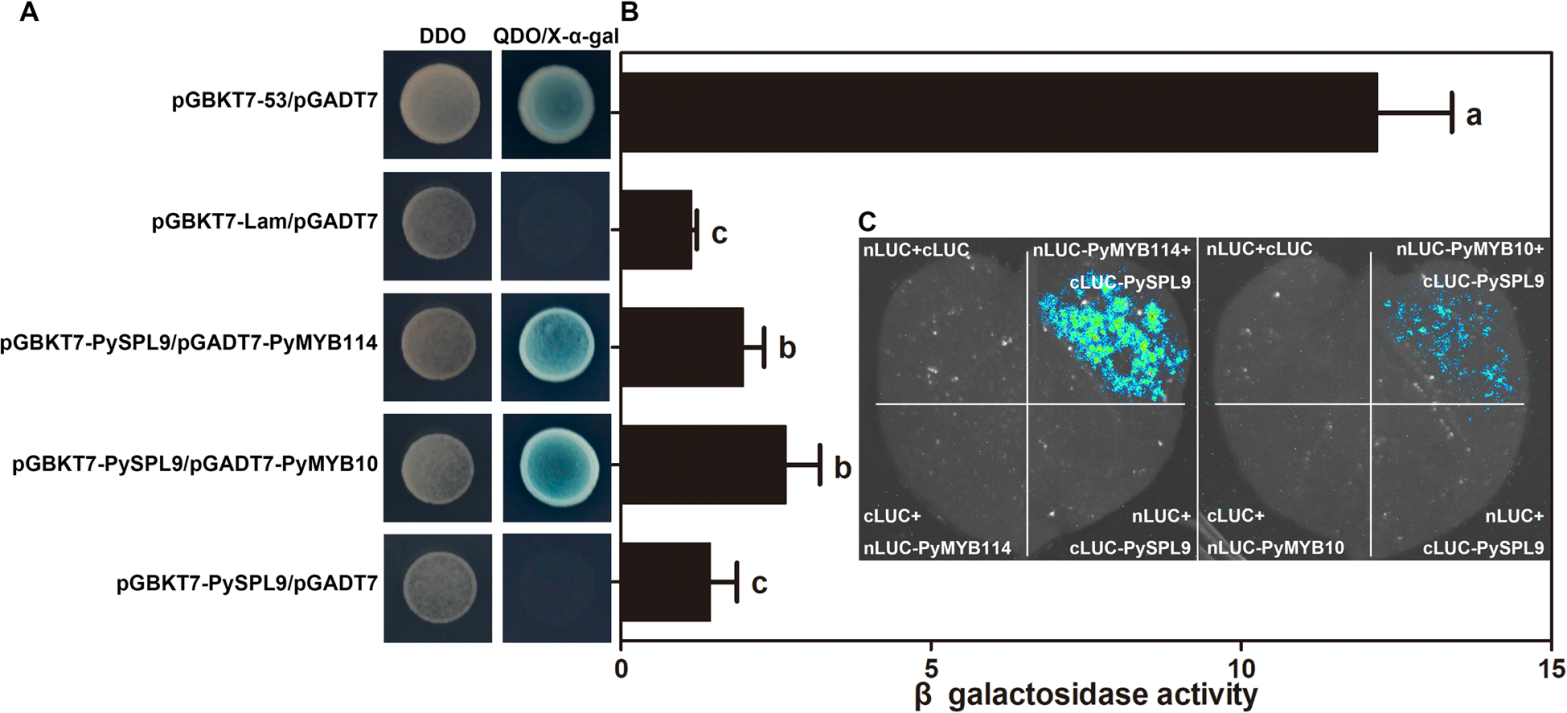
The interaction between *PymiR156a*-targeted PySPL9 and PyMYB114/10. **A** The interaction between PySPL9 and PyMYB114/10 verified by yeast two-hybrid assays. DDO: double dropout SD medium (lacking leucine and tryptophan). QDO/X-α-gal: quadruple dropout SD medium (lacking adenine, histidine, leucine and tryptophan) containing X-α-gal. **B** Detection of β galactosidase activity. **C** Firefly Luciferase Complementation Imaging (LCI) assay for protein-protein interactions

To explore the role of *PymiR156a*-targeted PySPL9 (homologous to *Arabidopsis* AtSPL9) during light-induced anthocyanin biosynthesis in pear skin, PySPL9 protein expression vectors were constructed in pGBKT7, and PyMYB114 and PyMYB10 were fused to pGADT7 to test interactions using the yeast two-hybrid assay (with Y2H Gold Yeast Strain). Our results indicated that PySPL9 interacted with both PyMYB114 and PyMYB10 (Fig. 5A). β-galactosidase assays (o-Nitrophenyl-β-D-galactoside, ONPG) were carried out to quantify and compare the strength of this protein-protein interaction. No significant difference in interaction intensity between PySPL9-PyMYB114 and PySPL9-PyMYB10 protein was observed (Fig. 5B), indicating similar stability of these two SPL-MYB protein complexes. The Firefly Luciferase Complementation Imaging (LCI) assay further confirmed the interaction of PySPL9-PyMYB114 and PySPL9-PyMYB10 (Fig. 5C).

In addition, we also compared the expression patterns of the complex members in bagged and debagged treatment, and found that the expression levels of *PyERF3*, *PybHLH3* and *PyWD40* in the skins from bagged fruits were relatively low (Fig. S6). Results are consistant with up-regulation of *PyPIF5* which promotes the accumulation of PySPL9 by suppression the expression of *PymiR156a*. In debagged fruits there is low expression of *PyPIF5* weakening its inhibitory effect on *PymiR156a* which would result in degradation of the target *PySPL9*. Transcripts of other complex members in the anthocyanin regulatory complex are upregulated to elevate expression of downstream anthocyanin related genes.

## Discussion

### The PyPIF5-*PymiR156a*-PySPL9-MYB114/10 module regulates light-induced anthocyanin biosynthesis in pear

Light is an important environmental factor that affects the growth and development of plants. The complex and sophisticated systems for sensing and response to light signals have been the target of long-term evolution (Chory and Wu 2001). PIFs, which play a central role by activating or repressing expression of downstream genes in light signaling during plant photomorphogenesis, work as a cellular signaling hub that integrates multiple signals to coordinate transcriptional networks (Shin et al. 2009; Leivar and Quail 2011; Pfeiffer et al. 2014). While, there are more than one *PIFs* among the differentially expressed genes (DEGs) in transcriptome data from bagged and debagged pear. A gene annotated as *PIF3* is up-regulated in the debagged fruit compared to the fruit still in the bag. As previous studies have shown that PIF3 act as a positive regulator during anthocyanin accumulation (Shin et al. 2007), and we got a similar result in our experiments (Fig. S7). Therefore, we focused on the role of down-regulated *PyPIF5* to discover and reveal differential regulatory mechanisms.

Some of these PIF-regulated networks include embryo development (Oh et al. 2004), seed germination (Leivar and Quail 2011; Oh et al. 2004), hypocotyl elongation (Monte et al. 2004), chlorophyll biosynthesis (Shin et al. 2009; Monte et al. 2004), shade avoidance (Xie et al. 2017; Nozue et al. 2011), and the circadian clock (Fujimori et al. 2004). In previous studies, it has been shown that PIF regulates shade avoidance by affecting *AtmiR156* in *Arabidopsis* (Xie et al. 2017). However, to date there has been no evidence to support that PIFs act as upstream regulators of miRNA involved in anthocyanin biosynthesis.

**Fig. 6:**
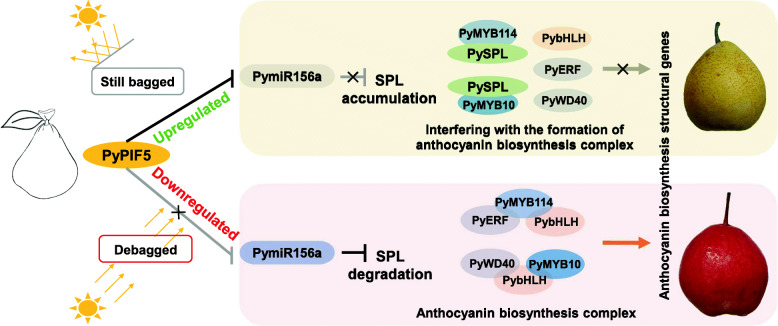
A negative regulatory model of PyPIF5 on *PymiR156a*-mediated light-induced anthocyanin biosynthesis in pear. In the light PyPIF5 does not maintain repression of the expression of *PymiR156a*, therefore *SPL* mRNA is cleaved, and SPL proteins decline in the complex

To investigate potential regulators, we performed cis-acting regulatory elements analysis of *PymiR156a* promoter. Multiple PIF-binding sites were found in *PymiR156a* promoter. PyPIF5 was identified based on differential expression analysis, while dual-luciferase assays and EMSA results indicated that PyPIF5 negatively regulates *PymiR156a* expression. Based on these results, we propose a putative model (Fig. 6). We found that PyPIF5 acts as an upstream transcription factor that directly binds to the G-box motif in *PymiR156a* promoter and suppresses its expression in bagged fruits (in the absence of light), thus relieves the cleavage effect of *PymiR156a* on the target *PySPL* genes. PySPL then accumulates to form heterodimers with crucial members of the anthocyanin biosynthesis regulatory complexes (PyMYB114 and PyMYB10), thus blocking the regulation of downstream genes that encode anthocyanin biosynthesis enzymes. In contrast, *PyPIF5* was significantly down-regulated when the fruits were re-exposed to natural light, and lost its inhibition of *PymiR156a.* Meanwhile, enough mature *PymiR156a* was produced and the targets were degraded thoroughly. In this way, the transcription complexes can be formed without being inhibited PySPL, thereby completing the regulation of anthocyanin biosynthesis genes (Fig. 6). From this data we reveal a direct regulatory link between two important regulators, upstream TFs and miRNAs, during light-induced anthocyanin biosynthesis in pear.

Besides that, there were other TFs might participate in this process based on cis-acting element analysis of *PymiR156a* promoter. In addition to PIF-binding sites, GATA-binding sites (GATA-motif), BBX-binding sites (GT1-motif), and MYB-binding sites (I-box) were also found in the *PymiR156a* promoter. We investigated the transcription level of these TFs, and found that not all of these corresponding motif-binding TFs showed a similar expression pattern as *PyPIF5* in bagged and debagged fruits (Fig. S8), which might be related to the complexity of light-induced anthocyanin regulation. In the current research we are not yet sure whether these TFs can bind to *PymiR156a* promoter and play regulatory effect, which needs further research. Considering the results of previous studies, other regulatory mechanisms (protein-protein interaction, DNA-protein interaction, other miRNA-target interaction, and epigenetic regulation etc.) also contribute significantly in the late anthocyanin biosynthesis and the final ripening stage. This might be the reason for the inconsistency of different expression *PyPIF5* and significant different expression *PymiR156a* at the 8th day. Therefore, we do not exclude the possibility that other TFs or regulatory mechanism have a regulatory effect on *PymiR156a*.

In summary, even though some progress has been made in the study of the regulation mechanism of light-induced anthocyanin biosynthesis for plant species, it remains to be determined how light response factor play a regulatory role during miRNA-mediated light-induced anthocyanin accumulation. In present study, we perfected regulatory mechanism of *PymiR156a* affecting anthocyanin biosynthesis through target genes, and pioneered an alternative PIF5-miRNA-SPL-MYB module in light-induced anthocyanin biosynthesis.

### Heterodimers of PySPL9 with PyMYB114 or PyMYB10 may co-exist in bagged fruits

MiR156 is an important miRNA family that plays roles in plant growth and development, metabolism regulation and stress response, and exerts biological functions through modulating target *SPL* genes (Cui et al. 2014; Cui et al. 2020; Gou et al. 2011; Miao et al. 2019). The SPL family is a plant specific transcription factor class with multiple functions. Gene family analysis of SPLs has been completed in diverse plant species, such as *Arabidopsis* (Cardon et al. 1999), apple (Li et al. 2013), grapevine (Wang et al. 2010), tomato (Salinas et al. 2012), rice (Xie et al. 2006) and cotton (Cai et al. 2018). Some members have been reported to be involved in anthocyanin biosynthesis in *Arabidopsis* (Gou et al. 2011), apple (Yang et al. 2019), peach (Zhou et al. 2015) and pear (Qian et al. 2017). In the present study, we identified that *PymiR156a* was significantly upregulated, while several *PySPLs* (Take *SPL9* as a typical representative) were significantly downregulated during light-induced anthocyanin accumulation (Fig. 3D), and this was caused by cleavage events driven by *PymiR156* on *PySPL* (Fig. 3G). In addition to *PySPL9*, *PySPL13,* which is also one of the predicted targets of *PymiR156a,* was also differentially expressed in Ox-miR156a inoculated red sector (Fig. S9), indicating the possible regulating function for *PymiR156a-PySPL13.* Comparatively, the *PySPL13* showed significant differences in two stages, except 8 days after debagging (Fig. 3E). We deduced the function of *PymiR156a* targeting *PySPL13* might involve in anthocyanin regulation, but not by activating *PySPL9* in all stages*.*

Interestingly, the expression of *SPL* family members in bagged fruits was generally lower than that of debagged fruits. This was contrary to the expression trend seen in apple where *SPL2* and *SPL33* activated anthocyanin biosynthesis (Yang et al. 2019). According to phylogenetic and qRT-PCR analysis (Fig. 3), apple SPL2 and SPL33 were not in the same cluster, and the expression pattern of the pear homologous genes were different, suggesting different and diverse functions of the SPL family between species.

It was known that the formation of an ERF/MYB/bHLH and MYB/bHLH/WD40 complex plays important roles in the anthocyanin biosynthesis in pear (Yao et al. 2017; Qian et al. 2017). We revealed that PySPL9 (a close homolog of *A. thaliana* AtSPL9) interacted with PyMYB114 or PyMYB10 (Fig. 5A), which are the central members of the anthocyanin biosynthesis regulatory complexes (Feng et al. 2010; Yao et al. 2017). These potentially form two independent heterodimers. In addition, we also evaluated the protein interaction strength in these two heterodimers by use of the β-galactosidase activity. No significant difference was observed between the two heterodimers, which indicated that they could coexist to function redundantly.

As two important transcriptional regulators, we have proved in vitro that PyMYB114 and PyMYB10 interact with the target PySPL of *PymiR156a* and proposed possible regulatory models, whether there is any interaction in pear requires further in vivo experimental verification.

We performed small RNA and transcriptome sequencing analysis with bagged and debagged pear fruits. The integrated analysis of DE-miRNAs and DEGs combined with experimental results revealed that a specific PIF, PyPIF5 works as a negative regulator of anthocyanin biosynthesis in the absence of light. PyPIF5 directly binds to the G-box motif in the promoter of *PymiR156a* to suppress its expression, which relieves the cleavage effect of *PymiR156a* on target *PySPL9*. The accumulated PySPL9 then forms heterodimers with PyMYB114 or PyMYB10 proteins. The regulation of MYBs related complex to downstream anthocyanin-related biosynthesis genes is therefore blocked. Our results complement the study of upstream regulation of miRNA towards understanding of the miRNA-mediated light-induced anthocyanin biosynthesis regulation network.

## Methods

### Plant materials and treatments

Red Chinese sand pear (cultivar “Yunhongyihao”) fruits were obtained from Yunnan Academy of Agricultural Sciences in Kunming, Yunnan, China. We bagged fruitlets at 35 days after full bloom with double-layered yellow-black paper bags [Kobayashi (Qingdao) Co., Ltd., China]. Half of the fruits were re-exposed to sunlight after the bags were removed at 10 days before harvest, and fruits were randomly sampled at 4 (D1), 8 (D2), and 10 (D3) days after bag removal (DABR). The fruits that remained bagged served as control samples (B1, B2 and B3). The skin from 10 individual fruits were pooled, instantly frozen in liquid nitrogen, and stored at − 80 °C for assays.

### Small-RNA sequencing and data analysis

A total amount of 3 μg total RNA from B1, B2, B3, D1, D2 and D3 (three biological replicates in per sample) was used for small RNA library construction. Sequencing libraries were constructed by NEBNext® Multiplex Small RNA Library Prep Set for Illumina® (NEB, USA). After library quality assessment and cluster generation, the qualified libraries were sequenced by an Illumina Hiseq 2500 platform with 50 bp single-end reads. After filtering, the remaining clean reads were used to search against miRBase database (Released 21, http://www.mirbase.org/) or align miRNA sequences in miRBase against reference genome for pear miRNAs identification. The available software miREvo (Wen et al. 2012) and mirdeep2 (Friedlander et al. 2011) were integrated to predict novel miRNA. psRobot_tar in psRobot (Wu et al. 2012) was used for the target gene prediction of miRNAs. DESeq R package (1.8.3) was used for differential expression analysis.

### RNA-sequencing and data analysis

A total amount of 1 μg RNA from B1, B2, B3, D1, D2 and D3 (three biological replicates in per sample) was used for the RNA sample preparations. Sequencing libraries were generated using NEBNext® Ultra™ RNA Library Prep Kit for Illumina® (NEB, USA). Illumina Hiseq platform was used for sequencing and 125 bp/150 bp paired-end reads were generated. Clean reads were obtained after filtering and removing. The Q20, Q30, GC-content, and also the duplication levels were calculated. All subsequent analyses were based on clean data with high quality. DESeq R package (1.18.0) was used for differential expression analysis, GO and KEGG enrichment analysis of DEGs were also performed.

### Expression profiles analysis

Total RNA was extracted using a modified CTAB method (Porebski et al. 1997). The total RNA sample was treated with DNase I to remove any contaminating genomic DNA, and then quantified.

For miRNA qRT-PCR (Quantitative reverse transcription PCR) analyses, miRNA first-Strand was synthesized using the Mir-X™ miRNA First-Strand Synthesis and SYBR® qRT-PCR Kit according to the instructions (638,313, Takara Biotechnology Co., Ltd). For target mRNA qRT-PCR analyses, first-strand cDNA was synthesized using TransScript® One-Step gDNA Removal and cDNA Synthesis SuperMix (AT311, Transgene, Beijing, China) (Pei et al. 2020).

The qPCR experiment was performed on LightCycler 480 Real-Time PCR instrument (Roche Diagnostics®, Basel, Switzerland). All reactions were performed with three replications, and *U6 snRNA* and *PyTUB* were respectively used as the internal control for miRNA and mRNA. Relative expression level of each gene was calculated using the 2^-ΔΔCp^ algorithm. The qPCR primers are listed in Supplementary Table S2.

### Assay of promoter activity and GUS staining

The expression vector consisting of the β-glucuronidase reporter gene (GUS) coding sequence driven by *PymiR156a* promoter was constructed by inserting the promoter into *Hind* III and *EcoR* I cloning sites of pCAMBIA0390::GUS vector by using the corresponding primers (Table S2). After confirmation by sequencing, the recombinant construct was transformed into *Agrobacterium tumefaciens* strain GV3101 for transient expression in *Nicotiana benthamiana* leaves through vacuum infiltration (Santos-Rosa et al. 2008). Then, the transformed leaves were cultivated at 25 °C for 24 h.

GUSBlue KIT (GT0391 & GT0392, Huayueyang Biotech Co., Ltd., Beijing, China) was used for histochemical GUS staining according to the instruction. Following staining (after 12 h of staining), the transformed leaves were washed with a graded ethanol series (70–90%) to wipe off chlorophyll until they turn transparent. Expression of pro-PymiR156a::GUS was observed and photographed using a digital camera.

### PyPIF5-his fusion protein expression and purification

The open reading frames of PyPIF5 were cloned into the pColdTF vector, which contained a Histidine (His) tag sequence, and the recombinant vector (pCold-PyPIF5) was then transformed into *Escherichia coli* BL21. The BL21 bacteria contained pCold-PyPIF5 was cultured at 37 °C (200 rpm) until OD_600_ ~ 0.6. After cooling, the liquid culture was subsequently treated with 1 mM (100 μL/100 mL) isopropyl β-D-1-thiogalactopyranoside (IPTG) to induce the PyPIF5-His fusion protein production (at 16 °C, 150 rpm for 24 h). The fusion protein was released from the cells by sonication and further purified with Ni-NTA His Bind Resin (Sangon Biotech. Co., Ltd., Shanghai, China). After elution with different concentration gradients of imidazole, the proteins were collected and detected by SDS-PAGE (Sodium dodecyl sulfate polyacrylamide gel electrophoresis).

### EMSA

EMSA was performed using biotin-labeled probes and the LightShift Chemiluminescent EMSA Kit (GS009, Beyotime, Shanghai, China). The *PymiR156a* promoter sequence containing G-box were used to generate the biotin-labeled (Bio-probe) and unlabeled probes (Cold-probe). In addition, biotin-labeled mutate-probe (Bio-Mut-probe) was generated by mutating recognition site (G-box motif) for competition analysis. Briefly, 500 ng of PyPIF5-His protein was incubated together with 20 nM biotin-labeled probes in 20 μL reaction mixtures (with 4 μL 5 × EMSA/Gel-Shift and Nuclease-Free Water for supplement the reaction volume) for 20 min at room temperature (~ 25 °C). Cold-probe or Mut-probe was added for the competition reactions.

### Overexpression and suppression vector construction for *PymiR156a* and agrobacterium-mediated transformations

To generate *PymiR156a* overexpression vector, a 300-bp stem-loop fragment of PymiR156 precursor was amplified by PCR from the pear genomic DNA and inserted into *pEASY*®-Blunt cloning vector (CB101, Transgene, Beijing, China). After monoclonal sequencing, the stem-loop fragment was inserted into the GFP vector, which carries green fluorescent protein and the selectable marker gene hygromycin phosphotransferase (*hph*).

For *PymiR156a* suppression vector, the short tandem target mimic (STTM) construction was referred to, as described by Tang et al. (2012). The STTM structure was constructed by PCR, and inserted into an intermediate vector (*pEASY*®-Blunt cloning vector, Transgene, Beijing, China), then introduced into the GFP vector through *Bam*H I and *Xba* I sites. The final expression vector GFP-Ox-*PymiR156*, GFP-STTM-*PymiR156* (Fig. S10) were transferred into *Agrobacterium tumefaciens* strain GV3101 using the freezing/heat shock method. And the suppression and overexpression vector of *PyPIF5* were also constructed by homologous recombination.

The transient transformation of pear skin and apple calli was as previously reported (Yao et al. 2017; An et al. 2017). *Arabidopsis thaliana* was used for *Agrobacterium*-mediated transformation following the procedure described by Clough and Bent (2018). Total anthocyanin content of the extracts was determined by spectrophotometric method (Vieira et al. 2018).

### Dual-luciferase reporter assay

*PymiR156a* targets were predicted by using the psRNATarget server (http://plantgrn.noble.org/psRNATarget/) (Dai and Zhao 2011). According to the prediction, the pGreenII Dual-Luciferase miRNA Target Expression Vector (Xue et al. 2019) derived from pGreenII 0800-LUC (Hellens et al. 2005) was used to quantitatively evaluate miRNA activity. For this evaluation, GFP-Ox-PymiR156, GFP-STTM-PymiR156 were used as effector, the reporter vector construction was as previously described by Xue et al. (2019), the predicted *PymiR156a* target and its flanking sequence (~ 100-bp upstream and downstream of target sequence) was amplified and inserted to 3′ terminal of the *LUC* gene (Fig. S10) by using ClonExpress® II One Step Cloning Kit (C112, Vazyme Biotech Co., Ltd., Nanjing, China).

*Nicotiana benthamiana*, which used for dual-luciferase reporter assay, were grown in a greenhouse with 16 h light at an average daily temperature of 25 °C. Four- to six-week-old *Nicotiana benthamiana* plants were infiltrated at the lower epidermal side of the leaves with needleless syringe. At least six leaves were infiltrated by one combination. Three days after infiltration, the leaves were used for dual-luciferase assay by the Dual-Luciferase Reporter Assay System (E1910, Promega Corporation, Madison, USA).

### Yeast two-hybrid and LCI assay

For yeast two-hybrid assay, the coding regions of *PyMYB114* and *PyMYB10*, *PySPL9* were PCR amplified from cDNAs and ligated to the pGADT7 and pGBKT7 vectors (Clontech, USA) to generate pGADT7-PyMYB114 and pGADT7-PyMYB10, and pGBKT7-PySPL9, respectively. The constructs were cotransformed into the yeast strain Y2H Gold (Clontech, Saint-Germain-en-Laye, France). Yeast transformation and liquid assay were conducted as described in the Yeast Protocols Handbook (Clontech). Transformants were grown on proper dropout plates containing X-α-gal (5-bromo-4-chloro-3-indolyl α-D-galactopyranoside) for positive interactions verification.

LCI assays were performed as previously described (Chen et al. 63). Briefly, *Agrobacterium* strain GV3101 carrying nLUC (nLUC-PyMYB114 and nLUC-PyMYB10) and cLUC (cLUC-PySPL9) construct combinations were infiltrated into tobacco leaves. LUC activities were detected using plant living imaging system (Princeton Instruments, USA).

### Supplementary Information

**Additional file 1 **: **Fig. S1.** Functional enrichment and overlap of predicted targets and DEGs.

**Additional file 2 **: **Fig. S2.** Promoter analysis of *PymiR156a* in transient assay using *GUS* reporter gene.

**Additional file 3 **: **Fig. S3.** Sequence logo of the APB and bHLH domain of PyPIF proteins.

**Additional file 4 **: **Fig. S4.** PyPIF5-His fusion protein and Probe sequence containing the G-box.

**Additional file 5 **: **Fig. S5.** Co-expression of *PymiR156a* and *PySPL9.*

**Additional file 6 **: **Fig. S6.** Expression pattern analysis of anthocyanin regulatory complex members.

**Additional file 7 **: **Fig. S7.** Expression pattern analysis of *PyPIF3* in begged and debagged fruits.

**Additional file 8 **: **Fig. S8.** Heatmap analysis of TFs that binding to corresponding cis-acting elements in *PymiR156a* promoter.

**Additional file 9 : Fig. S9.** Expression analysis of *PySPL9* and *PySPL13* in *Ox-miR156a* inoculated pear skin.

**Additional file 10 : Fig. S10.** Schematic representation of effector and reporter constructs used for dual-luciferase assays.

**Additional file 11 : Table S1.** Light-responsive cis-acting elements in promoter region of pre-*PymiR156a.*

**Additional file 12 : Table S2.** The primer sequences used in this study.

## Data Availability

The datasets during and/or analyzed during the current study available from the corresponding author on reasonable request.
