# Supplementary figures and images for: The PyPIF5-PymiR156a-PySPL9-PyMYB114/MYB10 module regulates light-induced anthocyanin biosynthesis in red pear
Source: Mol Hortic. 2021 Nov 3;1:14. doi: 10.1186/s43897-021-00018-5 (PMC10514999; doi:10.1186/s43897-021-00018-5)

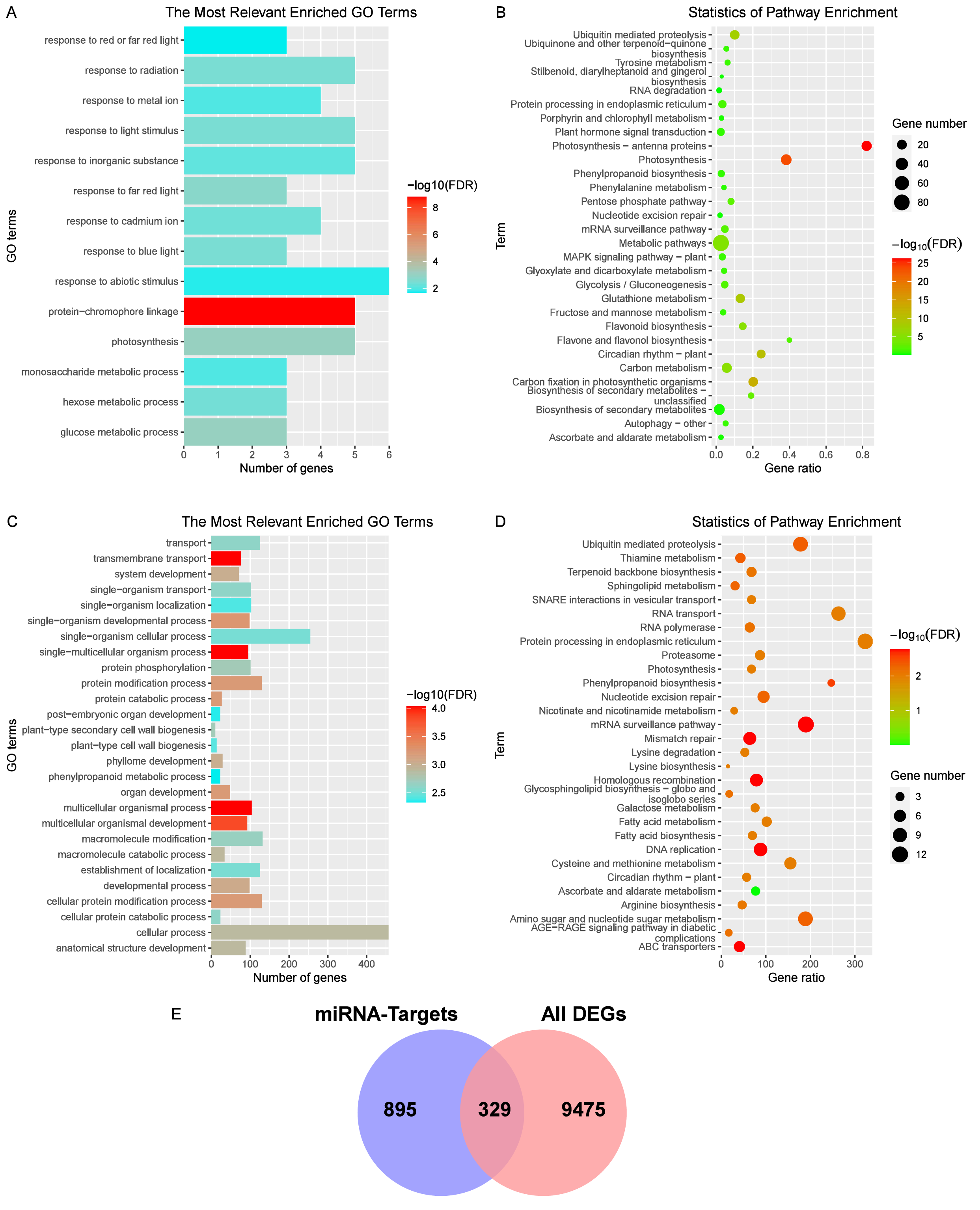

Supplement: Supplementary file 1 — Additional file 1 : Fig. S1. Functional enrichment and overlap of predicted targets and DEGs. [file 43897_2021_18_MOESM1_ESM.tif]

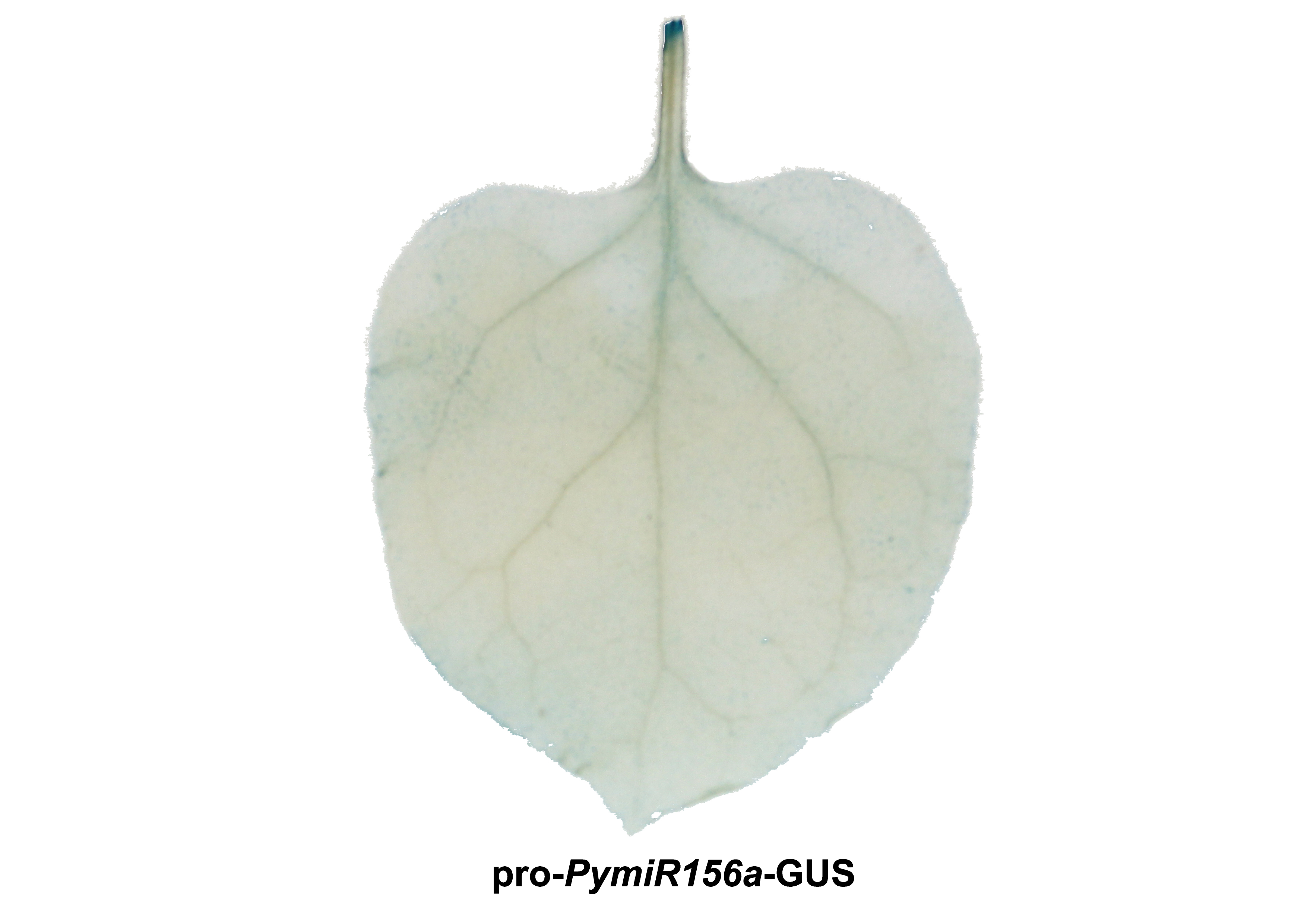

Supplement: Supplementary file 2 — Additional file 2 : Fig. S2. Promoter analysis of PymiR156a in transient assay using GUS reporter gene. [file 43897_2021_18_MOESM2_ESM.tif]

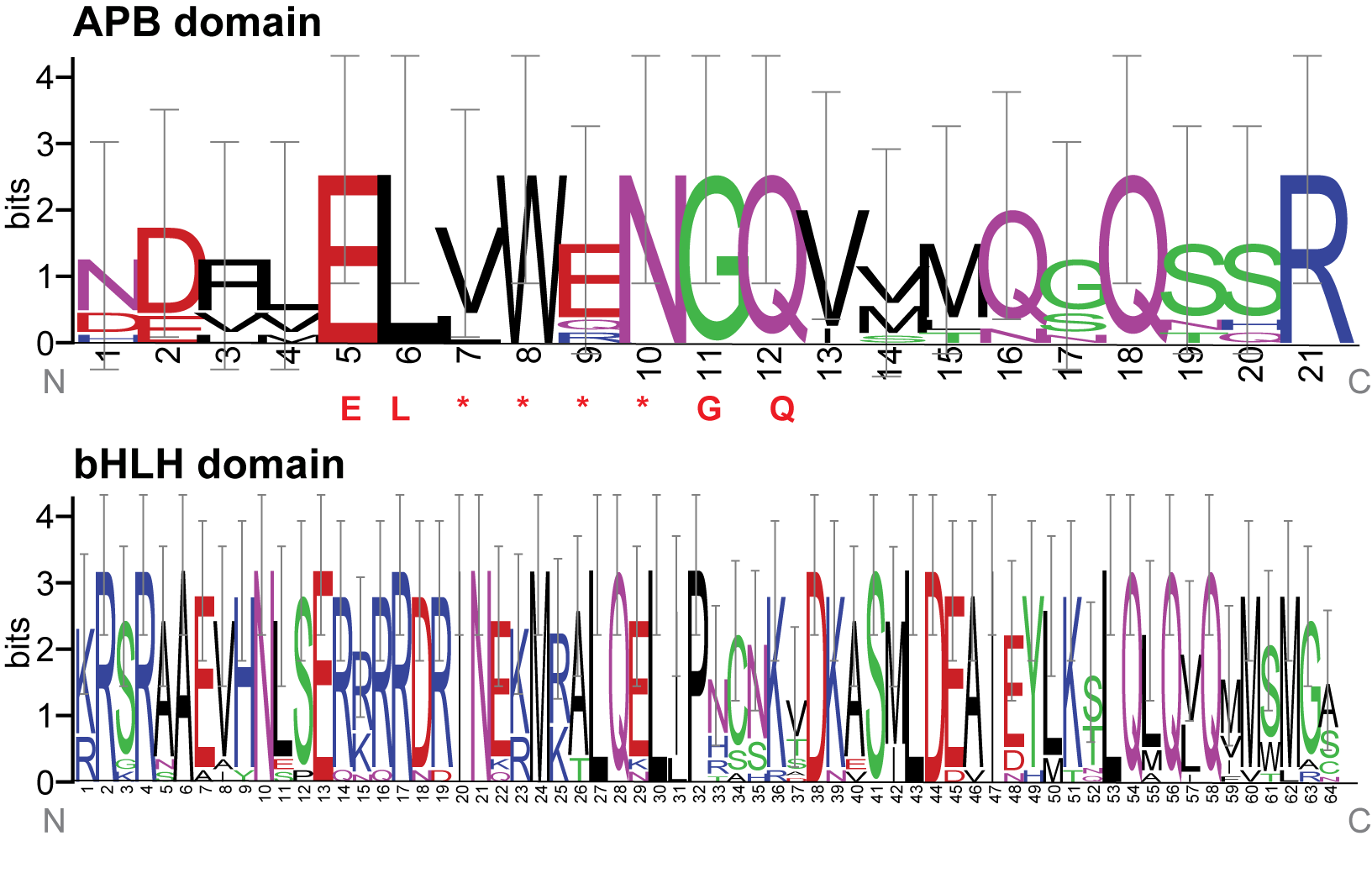

Supplement: Supplementary file 3 — Additional file 3 : Fig. S3. Sequence logo of the APB and bHLH domain of PyPIF proteins. [file 43897_2021_18_MOESM3_ESM.tif]

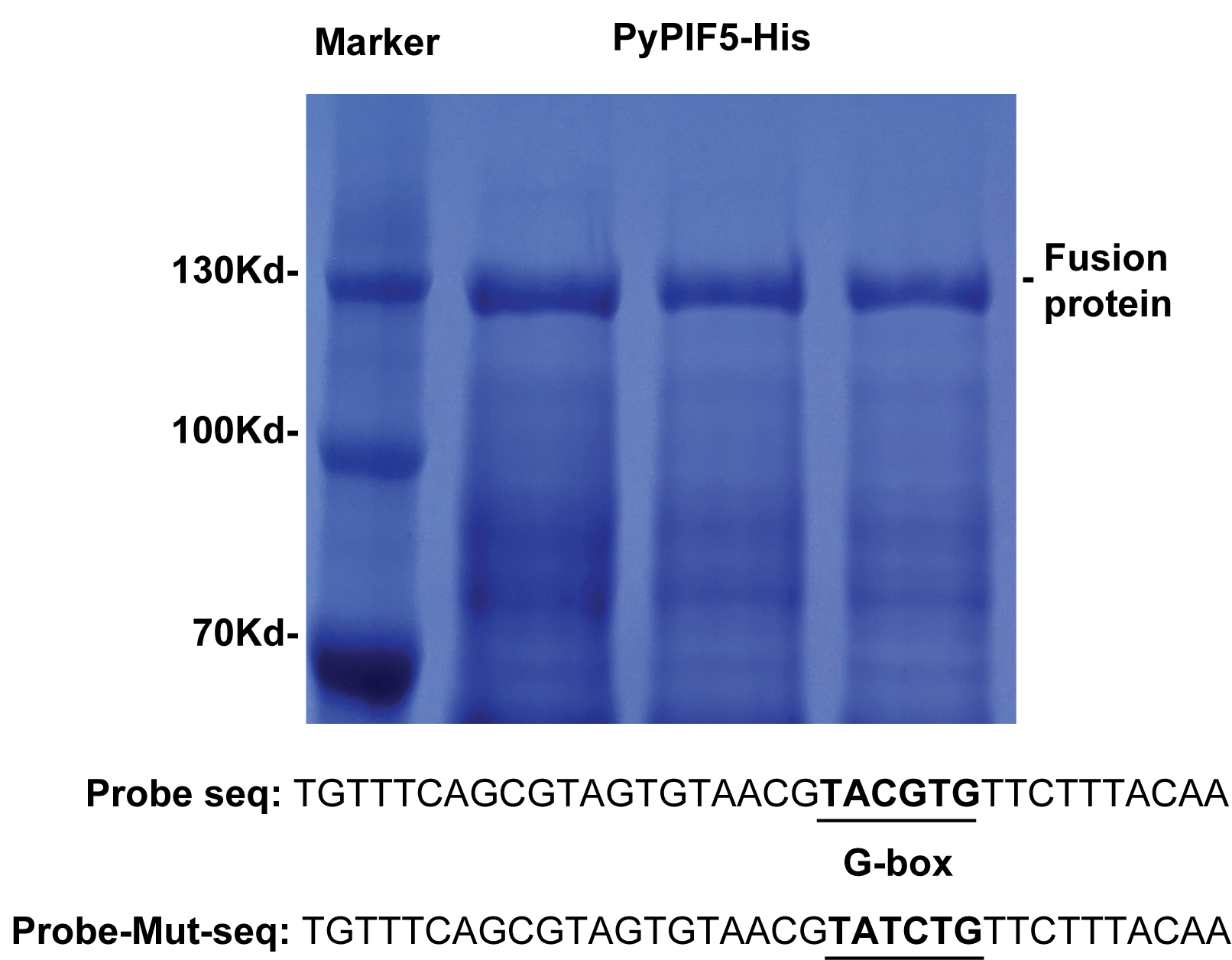

Supplement: Supplementary file 4 — Additional file 4 : Fig. S4. PyPIF5-His fusion protein and Probe sequence containing the G-box. [file 43897_2021_18_MOESM4_ESM.tif]

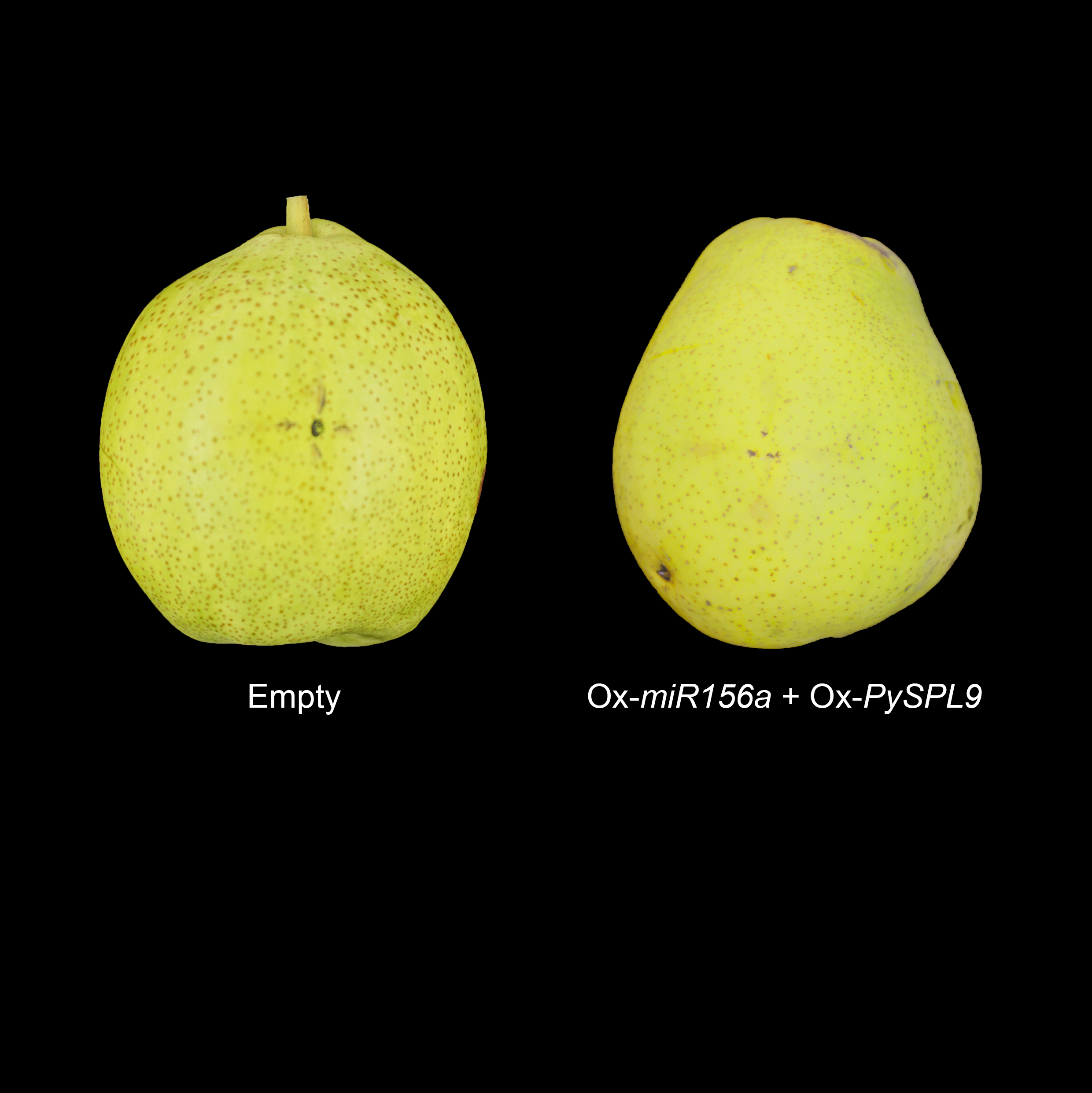

Supplement: Supplementary file 5 — Additional file 5 : Fig. S5. Co-expression of PymiR156a and PySPL9. [file 43897_2021_18_MOESM5_ESM.tif]

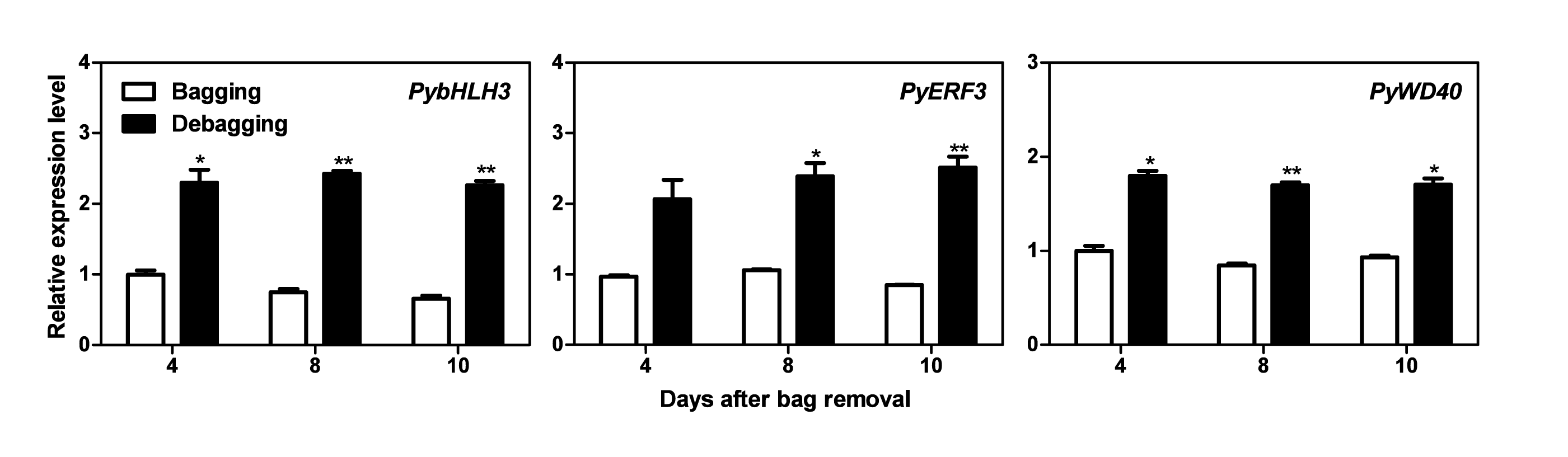

Supplement: Supplementary file 6 — Additional file 6 : Fig. S6. Expression pattern analysis of anthocyanin regulatory complex members. [file 43897_2021_18_MOESM6_ESM.tif]

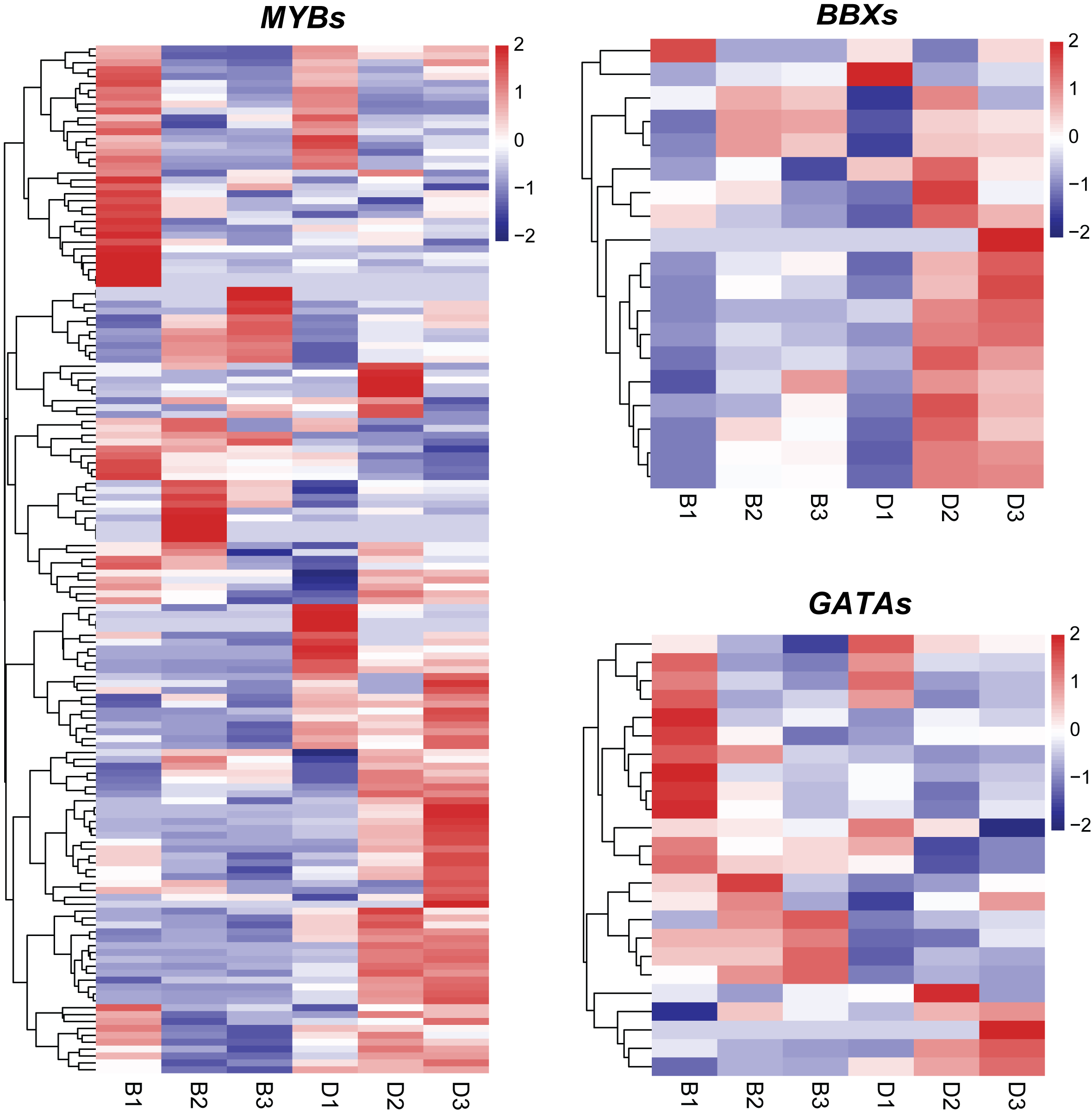

Supplement: Supplementary file 8 — Additional file 8 : Fig. S8. Heatmap analysis of TFs that binding to corresponding cis-acting elements in PymiR156a promoter. [file 43897_2021_18_MOESM8_ESM.tif]

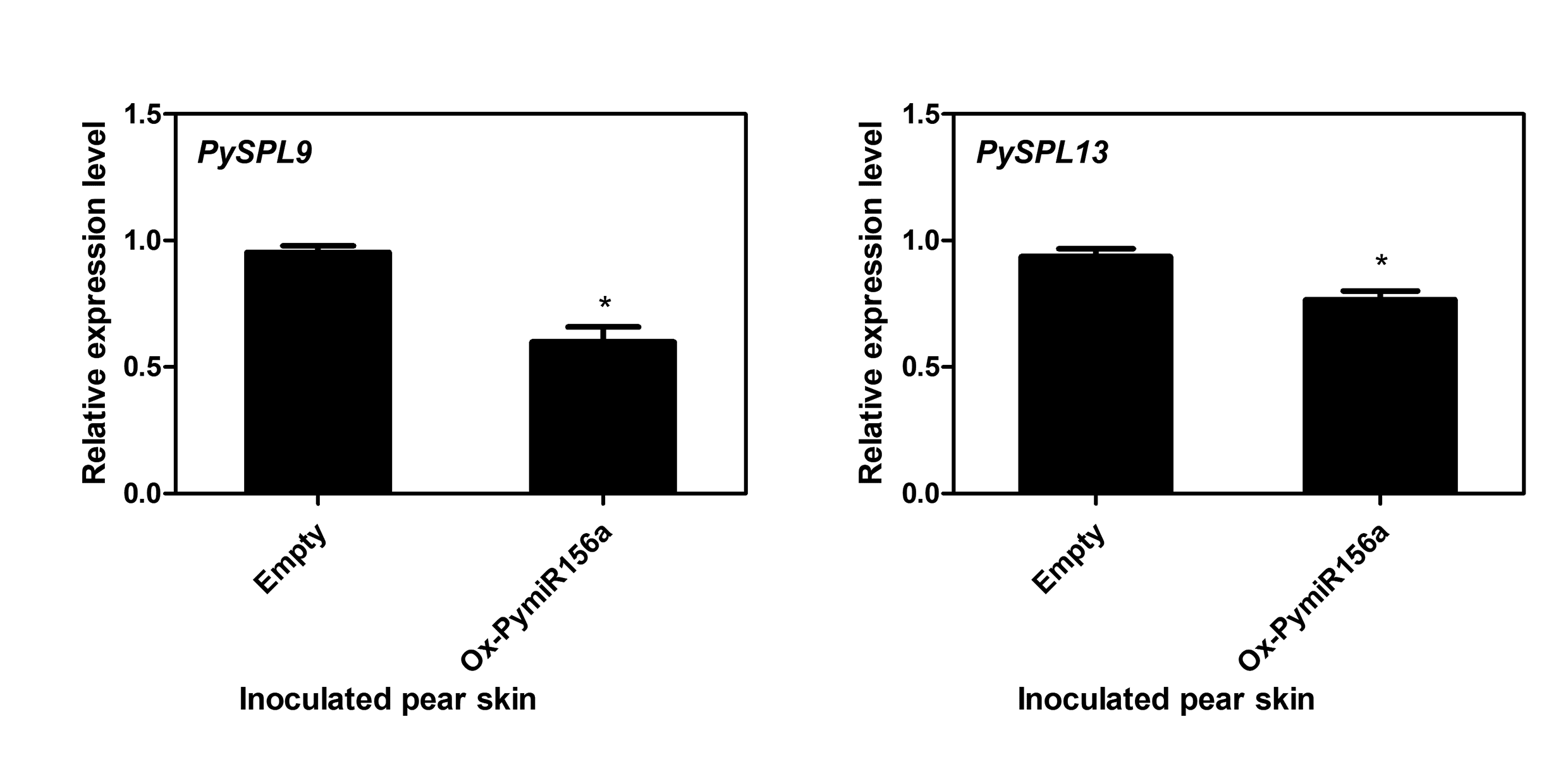

Supplement: Supplementary file 9 — Additional file 9 : Fig. S9. Expression analysis of PySPL9 and PySPL13 in Ox-miR156a inoculated pear skin. [file 43897_2021_18_MOESM9_ESM.tif]

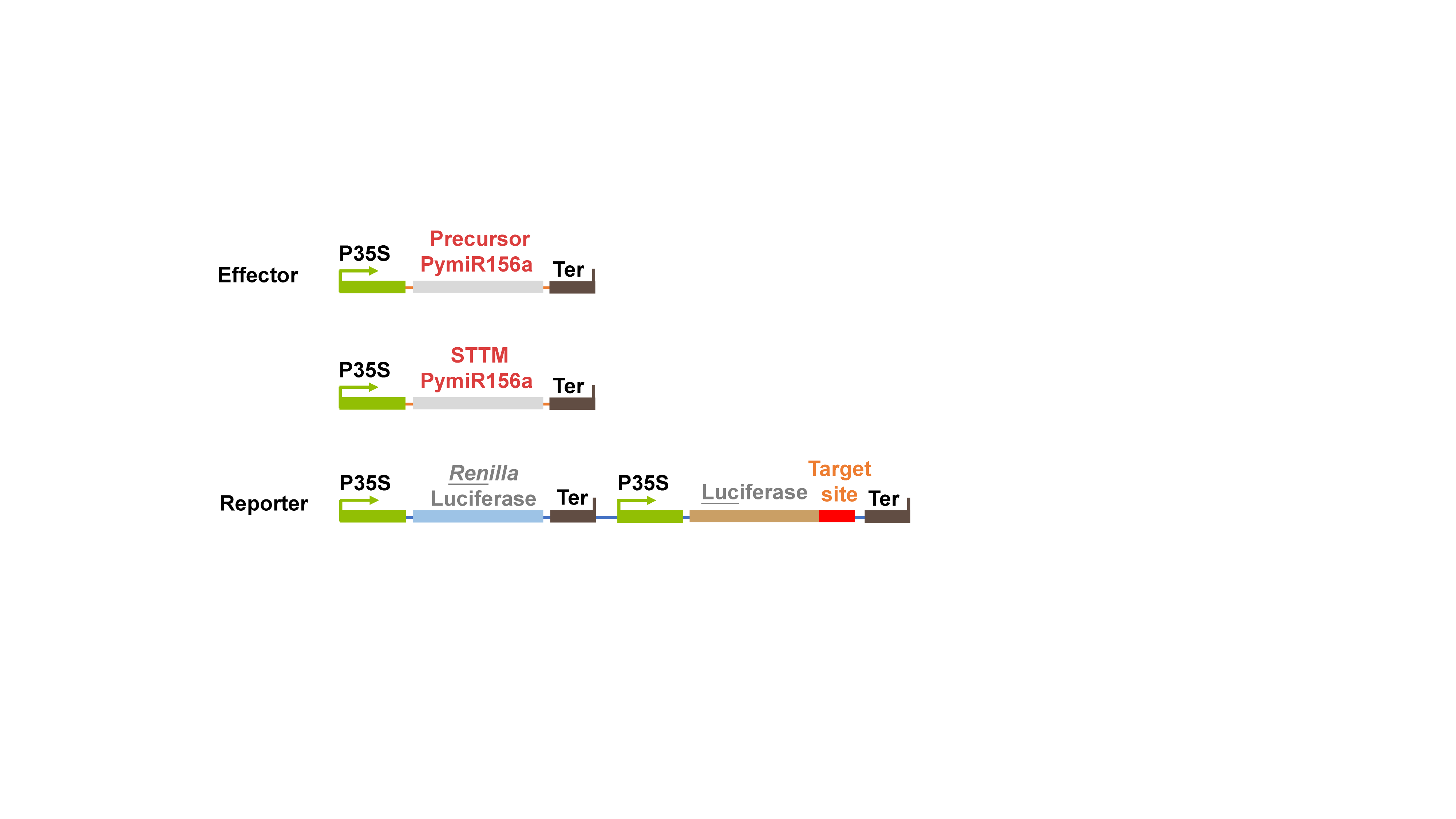

Supplement: Supplementary file 10 — Additional file 10 : Fig. S10. Schematic representation of effector and reporter constructs used for dual-luciferase assays. [file 43897_2021_18_MOESM10_ESM.tif]
